# Disruption of ER−mitochondria signalling in fronto-temporal dementia and related amyotrophic lateral sclerosis

**DOI:** 10.1038/s41419-017-0022-7

**Published:** 2018-02-28

**Authors:** Dawn H. W. Lau, Naomi Hartopp, Natalie J. Welsh, Sarah Mueller, Elizabeth B. Glennon, Gábor M. Mórotz, Ambra Annibali, Patricia Gomez-Suaga, Radu Stoica, Sebastien Paillusson, Christopher C. J. Miller

**Affiliations:** Department of Basic and Clinical Neuroscience, Institute of Psychiatry, Psychology and Neuroscience, King’s College London, 125 Coldharbour Lane Camberwell, London, SE5 9NU UK

## Abstract

Fronto-temporal dementia (FTD) and amyotrophic lateral sclerosis (ALS) are two related and incurable neurodegenerative diseases. Features of these diseases include pathological protein inclusions in affected neurons with TAR DNA-binding protein 43 (TDP-43), dipeptide repeat proteins derived from the *C9ORF72* gene, and fused in sarcoma (FUS) representing major constituent proteins in these inclusions. Mutations in *C9ORF72* and the genes encoding TDP-43 and FUS cause familial forms of FTD/ALS which provides evidence to link the pathology and genetics of these diseases. A large number of seemingly disparate physiological functions are damaged in FTD/ALS. However, many of these damaged functions are regulated by signalling between the endoplasmic reticulum and mitochondria, and this has stimulated investigations into the role of endoplasmic reticulum-mitochondria signalling in FTD/ALS disease processes. Here, we review progress on this topic.

## Facts

- ER−mitochondria signalling is disrupted by a number of FTD/ALS-linked insults. These include TDP-43, FUS, mutant SOD1, and loss of the Sigma-1 receptor.
- For TDP-43 and FUS this disruption involves breaking of the VAPB−PTPIP51 ER−mitochondria tethering proteins via activation of GSK3β.

## Open questions

- How do TDP-43 and FUS activate GSK3β?
- How does GSK3β regulate the VAPB−PTPIP51 interaction; is it via direct phosphorylation of one or both of these tethering proteins?
- Do other FTD/ALS insults also perturb ER−mitochondria contacts and signalling via disruption of the VAPB−PTPIP51 tethers? In particular, do pathogenic dipeptide repeat proteins derived from mutant *C9ORF72* damage the VAPB−PTPIP51 tethers and if so, does this involve GSK3β?
- Are ER−mitochondria contacts and the VAPB−PTPIP51 tethers damaged in human disease tissues?
- Is damage to ER−mitochondria signalling and the VAPB−PTPIP51 tethers an early pathogenic feature?
- Can ER−mitochondria signalling and the VAPB−PTPIP51 tethers be targeted pharmacologically?

## Fronto-temporal dementia and amyotrophic lateral sclerosis are related diseases

Fronto-temporal dementia (FTD), also known as fronto-temporal lobar degeneration, is characterised by neurodegeneration and neuronal loss in frontal and anterior temporal brain lobes. This leads to language impairment as well as behavioural and personality changes^1^. FTD is the second most common cause of presenile dementia after Alzheimer’s disease^2^. Amyotrophic lateral sclerosis (ALS) is the most common form of motor neuron disease. It involves degeneration of lower motor neurons in the brainstem and spinal cord, and of upper motor neurons in the motor cortex which together leads to progressive paralysis and muscle wasting. Survival time from symptom onset is only about 3 years^3^. There is no cure nor even effective disease-modifying treatments for either FTD or ALS.

Although originally classified as different diseases, FTD and ALS are now known to be clinically, genetically and pathologically linked. Approximately 15% of FTD patients display clinical ALS features and up to 15% of ALS patients develop symptoms consistent with a clinical definition of FTD^4,5^. Moreover, recent genetic and pathological studies have confirmed these links^2^.

Both FTD and ALS have strong genetic components, and mutations in a large number of genes are now known to be causative for inherited familial forms of these diseases. Indeed, there are now over 80 genes that have been linked to genetic forms of FTD/ALS and related motor neuron disorders^6^ (and see http://alsod.iop.kcl.ac.uk). Some of these mutant genes are more closely linked to either FTD or ALS. For example, mutations in *MAPT*, which encodes the microtubule-associated protein Tau, and *PGRN*, which encodes Progranulin, are linked almost exclusively to FTD. Likewise, mutations in *SOD1* that encodes the anti-oxidant enzyme Cu/Zn Superoxide dismutase-1 (SOD1) primarily causes ALS. However, mutations in a number of other genes cause dominantly inherited forms of both FTD and ALS. These include *TARDP* and *FUS/TLS* that encode the nucleic acid binding proteins TDP-43 and FUS, and *C9ORF72* whose encoded protein has been linked to autophagy^2^.

Interestingly, a number of these encoded proteins linked to familial FTD, ALS, and FTD/ALS also form pathologies of these diseases. Thus, Tau and SOD1 inclusions are seen in FTD and ALS respectively while TDP-43 and FUS inclusions form major pathologies in FTD/ALS^2,7^. Mutations in *C9ORF72* are causative for large numbers of familial FTD/ALS cases (up to about 30% FTD, 50% ALS and 80% FTD/ALS cases)^8–14^. The *C9ORF72* mutations involve expansion of an intronic hexanucleotide GGGGCC repeat and this repeat has been shown to be translated to generate dipeptide repeat proteins (DPRs) by a process termed repeat-associated non-ATG translation^15–17^. These DPRs are either poly- Gly-Pro, Pro-Ala, Gly-Ala, Pro-Arg or Gly-Arg and are deposited in FTD/ALS cases^15–17^. Some of these DPRs have been shown to be neurotoxic^18^. Interestingly, genetic forms of FTD/ALS caused by the *C9ORF72* mutations also often present with TDP-43 pathology and transgenic c9orf72 mice or mice expressing DPRs can develop TDP-43 pathology^7,19–23^. Together, these data suggest a link between DPRs and TDP-43. However, while DPR toxicity is the favoured disease mechanism for mutant *C9ORF72*, alternative hypotheses have been proposed. These involve haploinsufficiency and loss of c9orf72 function, and also the formation of RNA foci involving the GGGGCC repeat. These foci may sequester mRNA binding and/or other proteins to disrupt proper expression of heterologous genes^2^. Nevertheless, together these data show that there is some convergence of genetic and pathological phenotypes in FTD/ALS.

## FTD/ALS is characterised by damage to a variety of cellular functions and many of these are regulated by signalling between the ER and mitochondria

A number of physiological functions are perturbed in FTD/ALS^2,3,24,25^. These include damage to organelles and in particular mitochondria and the ER. Indeed, altered bioenergetics and activation of the unfolded protein response (UPR) are major features of FTD/ALS^26–28^. Disruption to Ca^2+^ homeostasis and changes to lipid metabolism are also seen in both diseases^29,30^. Axonal transport is a process by which proteins and organelles are transported to and from synapses and neurons are heavily dependent on this process. This is because most proteins are synthesised in cell bodies which then need to be transported to their final destinations including synapses; this transport can involve relatively long distances. Damage to axonal transport is a common feature of FTD/ALS^31,32^. Defective autophagy is also strongly implicated in FTD/ALS and some mutant genes linked to autophagy such as those encoding optineurin, ubiquilin-2, and SQSTM1/p62 are causative for familial forms of FTD/ALS^33,34^. Damage to autophagy may contribute to the failure of affected neurons to clear pathological protein aggregates in disease^27,35^. Mitophagy is specialised form of autophagy that involves the clearance of damaged mitochondria^36^. As stated above, damage to mitochondria contributes to FTD/ALS and so perturbations to mitophagy can lead to a failure to eliminate such damaged organelles. Finally, inflammatory responses are seen in FTD/ALS where reactive morphologies to astrocytes and microglia are prominent features along with the presence of inflammatory mediators and cytokines. It is generally believed that such inflammatory responses contribute to the disease process^37,38^. Indeed, anti-inflammatory agents can be protective in transgenic models of ALS^39^.

The biological conundrum is how so many apparently disparate physiological processes are damaged collectively. The therapeutic challenge is selecting which of these different processes to prioritise for drug discovery.

**Fig. 1:**
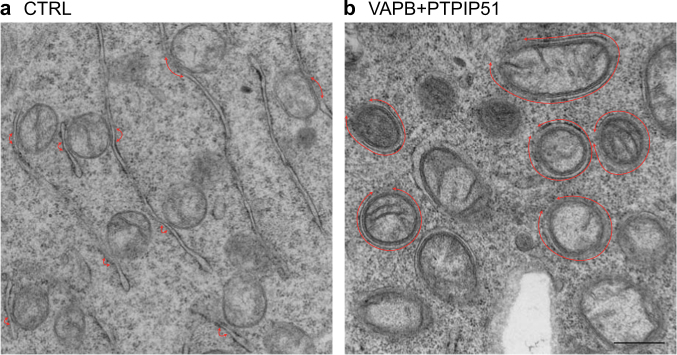
ER−mitochondria contacts in NSC-34 motor neuron cells Contacts are indicated with red arrows in control cells **a** and in cells transfected with the ER−mitochondria tethering proteins VAPB and PTPIP51 **b** Transfection of VAPB and PTPIP51 dramatically increases ER−mitochondria contacts. Scale bar = 500 nm

A number of recent studies have investigated signalling between ER and mitochondria in FTD/ALS. ER−mitochondria communication involves close physical contacts (10–30 nm distance) between the two organelles such that up to approximately 20% of the mitochondrial surface is tightly apposed to ER-membranes^40^ (Fig. 1a). These regions of ER are termed mitochondria-associated ER membranes (MAMs)^24,41–45^.

The reason for investigating ER−mitochondria signalling is that many of the damaged cell functions described above that characterise FTD/ALS are regulated by this signalling. Indeed, ER−mitochondria cross-talk is known to impact upon the following.

1. ER−mitochondria contacts facilitate phospholipid exchange between the two organelles. This is important as the enzymes that synthesise certain lipids are present in either organelle and so precursor exchange is required for the production of these lipids^24,41–45^. Indeed, MAMs have been shown to be a specialised type of lipid raft (also known as detergent-resistant membranes)^46^.
2. ER−mitochondria contacts facilitate Ca^2+^ exchange between the two organelles and in particular uptake of Ca^2+^ by mitochondria following its release from ER stores via inositol 1,4,5-trisphosphate (IP3) receptors. Such Ca^2+^ uptake is required by mitochondria for generating ATP via the tricarboxylic acid cycle since several mitochondrial dehydrogenases are Ca^2+^ regulated^47^. However, excessive uptake of Ca^2+^ by mitochondria can lead to opening of the mitochondrial permeability transition pore and signalling for apoptosis^24,41–45^.
3. ER−mitochondria contacts are required for mitochondrial biogenesis since mitochondrial fission occurs at contact sites and mitochondrial DNA synthesis is regulated by these contacts^48–50^.
4. ER−mitochondria contacts regulate intracellular trafficking of both mitochondria and ER since a proportion of ER is co-transported with mitochondria through cells^51^. Moreover, the Ca^2+^ sensor mitochondrial Rho GTPase (Miro), which mediates attachment of mitochondria to kinesin-1 motors for transport, localises to ER−mitochondria contact sites^52–55^. In neurons, this trafficking includes axonal transport.
5. ER−mitochondria contacts are linked to autophagy^56–61^. Notably, several groups have shown that delivery of Ca^2+^ from ER stores to mitochondria at MAM regulates autophagosome formation^61–69^.
6. ER−mitochondria contacts are linked to ER stress and the UPR. Several ER protein folding chaperones are present in MAM, disrupting ER-mitochondria contacts induces the UPR, and chemical induction of the UPR increases ER-mitochondria associations^70–72^. Moreover, vesicle-associated membrane protein associated-protein B (VAPB), which functions as an ER−mitochondria tethering protein (see below), is strongly linked to ER stress responses^73,74^.
7. ER−mitochondria signalling is linked to formation of the inflammasome, a multiprotein complex involved in the initiation of inflammatory processes and in particular, proteolytic maturation of the pro-inflammatory cytokine interleukin-1β. Notably, mitochondria-derived reactive oxygen species induce recruitment of the NOD-like receptor NLRP3, a key component of the inflammasome to MAM^75^.

Thus, damage to ER−mitochondria signalling represents a plausible route for explaining many pathological features of FTD/ALS.

## ER−mitochondria tethering proteins

**Fig. 2:**
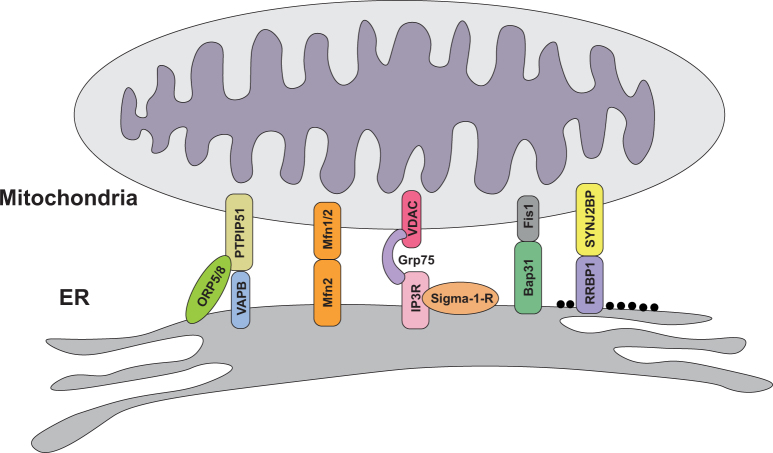
Proposed ER−mitochondria tethering and/or regulator proteins in vertebrates

Crucial to understanding both the normal roles of ER−mitochondria signalling and any abnormal role in disease is knowledge of the mechanisms by which regions of ER are recruited to mitochondria. It is now generally accepted that this recruitment involves scaffolding proteins that function to tether the two organelles. A number of tethering proteins have now been identified (Fig. 2). In yeast, proteins of the ER−mitochondria encounter structure (ERMES) function to connect the two organelles but ERMES proteins are yeast specific and no homologues have been found in mammals^76^. In mammals, the interaction between ER-located IP3 receptors and the mitochondrial voltage-dependent anion channel (VDAC) via Grp75 was originally proposed as a functional tether but loss of IP3 receptors does not affect ER−mitochondria contacts, which argues against a direct tethering role^40^. Homo- and heterotypic interactions between ER-located mitofusin-2 and mitochondrial mitofusin-1/2 have also been proposed as tethers^77,78^ but other groups have disputed these findings (see Filadi et al.^41^ for further discussion on this topic).

**Fig. 3:**
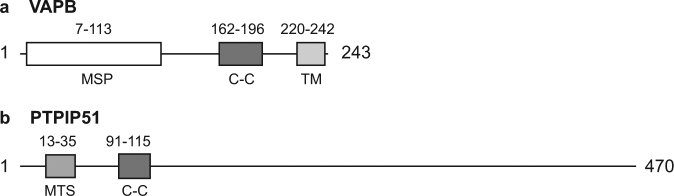
Domain structures of the ER−mitochondria tethering proteins VAPB and PTPIP51 **a** VAPB contains a major sperm protein (MSP) domain, a coiled-coil domain (C-C), and a transmembrane (TM) domain at the C-terminus. **b** PTPIP51 contains a mitochondrial targeting sequence (MTS) and a coiled-coil domain

More recently, binding of the integral ER protein VAPB and the outer mitochondrial membrane protein, protein tyrosine phosphatase interacting protein 51 (PTPIP51) has been shown to tether ER with mitochondria^79,80^ (see Fig. 3 for VAPB and PTPIP51 domain structures). VAPB binds to PTPIP51 in many different biochemical assays and modulating VAPB and PTPIP51 expression induces appropriate changes in ER−mitochondria contacts (Fig. 1b). Moreover, manipulating VAPB or PTPIP51 expression alters Ca^2+^ exchange between the two organelles which is a physiological readout of ER−mitochondria contacts^79–81^. Others have now replicated and extended these findings^82–86^. Thus, PTPIP51 may also interact with the oxysterol-binding protein-related proteins ORP5 and ORP8 to regulate ER−mitochondria contacts^83^.

Finally, the tail-anchored, PDZ-domain-containing outer mitochondrial membrane protein SYNJ2BP was shown to bind to the ribosome-binding protein 1 and this interaction may act to selectively mediate signalling between mitochondria and rough ER^87^. A number of other proteins have also been linked to ER−mitochondria signalling, including B-cell receptor-associated protein 31 (Bap31) and fission protein 1 (Fis1), FUN14 domain-containing protein 1 (FUNDC1) and calnexin, and phosphofurin acid cluster sorting protein 2 (PACS2), but whether these are bonafide tethering proteins or regulators of ER−mitochondria signalling is unclear^24,41,59,70,88^.

## ER−mitochondria signalling and the VAPB-PTPIP51 tethers are disrupted in FTD/ALS

**Fig. 4:**
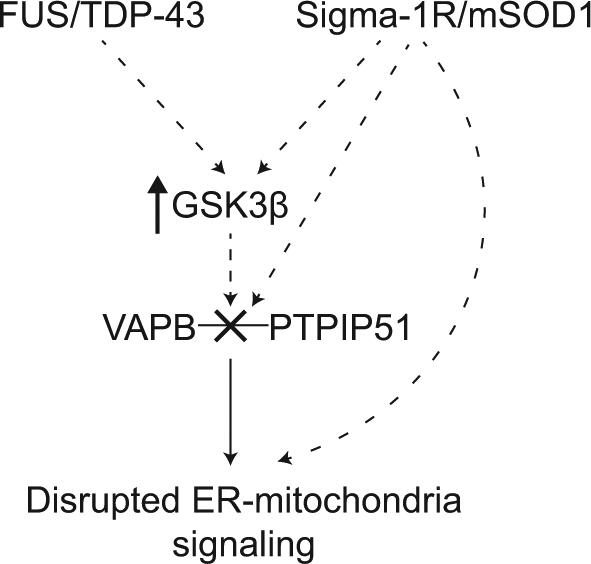
Model showing how different FTD/ALS insults disrupt ER−mitochondria signalling TDP-43 and FUS activate GSK3β which then leads to breaking of the VAPB−PTPIP51 tethering proteins, loosening of ER−mitochondria contacts and disruption to signalling. The mechanisms by which TDP-43 and FUS activate GSK3β and how GSK3β influences the VAPB−PTPIP51 interaction are not known. GSK3β may phosphorylate VAPB and/or PTPIP51 to regulate directly their binding or alter binding indirectly via other molecules. The molecular mechanisms linking the Sigma-1 receptor and mutant SOD1 to ER-mitochondria communication are less well characterised. They may alter ER-mitochondria signalling via GSK3β and the VAPB−PTPIP51 tethers or via other routes, including direct effects on the tethers

A number of studies have now investigated how FTD/ALS insults affect ER−mitochondria signalling. Both TDP-43 and FUS have been shown to disrupt ER−mitochondria interactions and this is associated with decreased binding of VAPB to PTPIP51^80,89^. TDP-43 and FUS also perturb Ca^2+^ exchange between ER and mitochondria (which is consistent with a loosening of ER−mitochondria associations) and mitochondrial ATP production which is dependent upon this Ca^2+^ exchange^80,89^. Moreover, the effects of TDP-43 and FUS on ER−mitochondria contacts and the VAPB−PTPIP51 tethers are linked to activation of glycogen synthase kinase-3β (GSK3β) (Fig. 4)^80,89^. GSK3β activation disrupts binding of VAPB to PTPIP51 and GSK3β inhibitors correct FUS induced damage to ER−mitochondria signalling^80,89^. GSK3β is strongly associated with neurodegenerative disease. For example, GSK3β is activated in induced pluripotent stem cell neurons derived from Alzheimer’s disease patients and regulates Aβ production, and phosphorylates Tau so that it resembles that seen in dementia^90–92^.

Other studies have linked loss-of-function mutations in the Sigma-1 receptor to familial FTD, ALS and other forms of motor neuron disease^93–99^. The Sigma-1 receptor is an ER protein enriched in MAM that facilitates IP3 receptor-mediated delivery of Ca^2+^ from ER stores to mitochondria; as such the Sigma-1 receptor enhances mitochondrial ATP production^100–102^. The Sigma-1 receptor gene resides on chromosome 9 and it has been suggested that the disease-causing mutations in some of these families may involve *C9ORF72* (which also resides on chromosome 9) and not the mutant Sigma-1 receptor^103^. However, further mutations in the Sigma-1 receptor have been linked to FTD and motor neuron disease and some of these have formally excluded the involvement of mutant *C9ORF72*^93,94,97,98^. Moreover, a variety of experimental studies have provided mechanistic data to link loss of the Sigma-1 receptor to FTD/ALS. Firstly, Sigma-1 receptor knockout mice display features of ALS. Secondly, loss of Sigma-1 receptor exacerbates disease in other transgenic mouse models of ALS and can induce features of FTD/ALS in cellular models. Finally, Sigma-1 receptor agonists have proved beneficial in some models of FTD/ALS^93,94,104–109^. Notably, disease mutant Sigma-1 receptor variants and loss of the Sigma-1 receptor have all been shown to reduce ER−mitochondria contacts and signalling although the mechanisms underlying these effects are not known (Fig. 4)^93,94,98^. Finally, ALS mutant SOD1 has also been shown to reduce ER−mitochondria contacts and signalling, and this is linked to a selective loss of the Sigma-1 receptor from MAM^94^. Thus, four different FTD/ALS-linked genetic insults, TDP-43, FUS, the Sigma-1 receptor and mutant SOD1, have all been shown to disrupt ER−mitochondria contacts and signalling and where investigated (TDP-43 and FUS) this involves breaking of the VAPB−PTPIP51 tethers^80,89,93,94,98^.

Interestingly, mutations in VAPB also cause some forms of ALS and related motor neuron diseases^2,3^. The best characterised mutant involves a proline to serine substitution at amino acid 56 VAPB-P56S^110^. Compared to wild-type, VAPB-P56S displays increased binding to PTPIP51^79^. This is in apparent contrast to other FTD/ALS mutant proteins which reduce ER−mitochondria contacts and the VAPB−PTPIP51 interaction^80,89,93,94,98^. However, VAPB-P56S expression is markedly lower than wild-type and also reduces total VAPB levels^73,111^. Thus, despite its increased binding to PTPIP51, the overall effect of VAPB-P56S may be to reduce ER−mitochondria contacts. Interestingly, reduced levels of VAPB are seen in sporadic ALS patients^112^.

## Is damage to ER−mitochondria signalling and MAM a 'driver' of disease, or a response to damage of other physiological processes?

The above studies that demonstrate damage to ER−mitochondria signalling in FTD/ALS provide a plausible mechanism for explaining many disease features. Thus, primary insults such as TDP-43, FUS, loss of the Sigma-1 receptor and mutant SOD1 may damage ER−mitochondria signalling which in turn perturbs other downstream cellular functions such as Ca^2+^ homeostasis, lipid metabolism, axonal transport, mitochondrial function and ER stress. In this scenario, damage to ER−mitochondria signalling represents a driver of the disease process and therapeutic correction of this damage may correct many disease features (Fig. 4). However, an alternative possibility is that alterations to ER−mitochondria signalling in disease represent a physiological response to other damaged features. Clearly, if targeting ER−mitochondria signalling is to be a valid drug target for FTD/ALS, then it is important to properly discriminate between these possibilities.

A number of lines of evidence suggest that disruption to ER−mitochondria signalling is a driver of disease. Firstly, mutations in *VAPB* cause some familial forms of ALS and these mutants reduce VAPB levels; selective reduction of VAPB is also seen in spinal cords of sporadic ALS patients^110–112^. Such loss of VAPB impairs ER−mitochondria communication^79,80^. Secondly, mutant loss of the Sigma-1 receptor, a key MAM protein also causes familial FTD/ALS and enhancing Sigma-1 receptor function is protective in FTD/ALS models^93,94,98^. Thirdly, overexpression of VAPB to restore ER−mitochondria tethering and signalling^61,80^ is protective in mutant SOD1 transgenic mice^113^. Finally, Parkinson’s disease-related α-synuclein disrupts the VAPB−PTPIP51 tethers and overexpression of VAPB to correct this disruption also corrects α-synuclein-linked damage to Ca^2+^ homeostasis^114^.

Future studies to address these issues further will involve determining whether ER−mitochondria contacts are perturbed in human FTD/ALS tissues and whether any damage is an early disease feature; early pathogenic changes are believed to be the most important. However, such studies are likely to be compromised by the quality of preservation in most post-mortem tissue. Utilising neurons derived from induced pluripotent stem cells carrying FTD/ALS associated mutations provides an alternative route. In a complementary fashion, determining whether damaged ER-mitochondria contacts and signalling are early pathogenic events in transgenic mouse models of FTD/ALS will be of major value. Finally, investigating whether experimental correction of damaged ER-mitochondria contacts corrects other disease features will provide evidence as to whether targeting the ER-mitochondria axis is a valid drug target for FTD/ALS.

## References

[1] Sieben A (2012). The genetics and neuropathology of frontotemporal lobar degeneration. Acta Neuropathol..

[2] Ling SC, Polymenidou M, Cleveland DW (2013). Converging mechanisms in ALS and FTD: disrupted RNA and protein homeostasis. Neuron.

[3] Ferraiuolo L, Kirby J, Grierson AJ, Sendtner M, Shaw PJ (2011). Molecular pathways of motor neuron injury in amyotrophic lateral sclerosis. Nat. Rev. Neurol..

[4] Ringholz GM (2005). Prevalence and patterns of cognitive impairment in sporadic ALS. Neurology.

[5] Wheaton MW (2007). Cognitive impairment in familial ALS. Neurology.

[6] Abel O, Powell JF, Andersen PM, Al-Chalabi A (2012). ALSoD: a user-friendly online bioinformatics tool for amyotrophic lateral sclerosis genetics. Hum. Mutat..

[7] Spires-Jones TL, Attems J, Thal DR (2017). Interactions of pathological proteins in neurodegenerative diseases. Acta Neuropathol..

[8] Boeve BF (2012). Characterization of frontotemporal dementia and/or amyotrophic lateral sclerosis associated with the GGGGCC repeat expansion in C9ORF72. Brain.

[9] Chio A (2012). Clinical characteristics of patients with familial amyotrophic lateral sclerosis carrying the pathogenic GGGGCC hexanucleotide repeat expansion of C9ORF72. Brain.

[10] Cooper-Knock J (2012). Clinico-pathological features in amyotrophic lateral sclerosis with expansions in C9ORF72. Brain.

[11] Hsiung GY (2012). Clinical and pathological features of familial frontotemporal dementia caused by C9ORF72 mutation on chromosome 9p. Brain.

[12] Mahoney CJ (2012). Frontotemporal dementia with the C9ORF72 hexanucleotide repeat expansion: clinical, neuroanatomical and neuropathological features. Brain.

[13] Simon-Sanchez J (2012). The clinical and pathological phenotype of C9orf72 hexanucleotide repeat expansions. Brain.

[14] Snowden, J. S. et al. Distinct clinical and pathological characteristics of frontotemporal dementia associated with C9ORF72 mutations. Brain 135, 693–708 (2012). 10.1093/brain/awr355 PMC3286329 22300873

[15] Mori K (2013). The C9orf72GGGGCC repeat is translated into aggregating dipeptide-repeat proteins in FTLD/ALS. Science (New York, NY).

[16] Mori K (2013). Bidirectional transcripts of the expanded C9orf72hexanucleotide repeat are translated into aggregating dipeptide repeat proteins. Acta Neuropathol..

[17] Ash PE (2013). Unconventional translation of C9ORF72 GGGGCC expansion generates insoluble polypeptides specific to c9FTD/ALS. Neuron.

[18] Mizielinska S (2014). C9orf72 repeat expansions cause neurodegeneration in Drosophila through arginine-rich proteins. Science (New York, NY).

[19] Al-Sarraj S (2011). p62 positive, TDP-43 negative, neuronal cytoplasmic and intranuclear inclusions in the cerebellum and hippocampus define the pathology of C9orf72-linked FTLD and MND/ALS. Acta Neuropathol..

[20] Liu Y (2016). C9orf72 BAC mouse model with motor deficits and neurodegenerative features of ALS/FTD. Neuron.

[21] Mackenzie IR, Frick P, Neumann M (2014). The neuropathology associated with repeat expansions in the C9ORF72 gene. Acta Neuropathol..

[22] Chew J (2015). C9ORF72 repeat expansions in mice cause TDP-43 pathology, neuronal loss, and behavioral deficits. Science (New York, NY).

[23] Mackenzie IR, Neumann M (2017). Reappraisal of TDP-43 pathology in FTLD-U subtypes. Acta Neuropathol..

[24] Paillusson S (2016). There’s something wrong with my MAM; the ER-mitochondria axis and neurodegenerative diseases. Trends Neurosci..

[25] Krols M (2016). Mitochondria-associated membranes as hubs for neurodegeneration. Acta Neuropathol..

[26] Cozzolino M, Ferri A, Valle C, Carri MT (2013). Mitochondria and ALS: implications from novel genes and pathways. Mol. Cell Neurosci..

[27] Cai Y (2016). Interplay of endoplasmic reticulum stress and autophagy in neurodegenerative disorders. Autophagy.

[28] Kanekura K, Suzuki H, Aiso S, Matsuoka M (2009). ER stress and unfolded protein response in amyotrophic lateral sclerosis. Mol. Neurobiol..

[29] Grosskreutz J, Van Den Bosch L, Keller BU (2010). Calcium dysregulation in amyotrophic lateral sclerosis. Cell Calcium.

[30] Schmitt F, Hussain G, Dupuis L, Loeffler JP, Henriques A (2014). A plural role for lipids in motor neuron diseases: energy, signaling and structure. Front. Cell Neurosci..

[31] Millecamps S, Julien JP (2013). Axonal transport deficits and neurodegenerative diseases. Nat. Rev. Neurosci..

[32] De Vos KJ, Grierson AJ, Ackerley S, Miller CCJ (2008). Role of axonal transport in neurodegenerative diseases. Annu. Rev. Neurosci..

[33] Majcher V, Goode A, James V, Layfield R (2015). Autophagy receptor defects and ALS-FTLD. Mol. Cell Neurosci..

[34] Markovinovic A (2017). Optineurin in amyotrophic lateral sclerosis: multifunctional adaptor protein at the crossroads of different neuroprotective mechanisms. Prog. Neurobiol..

[35] Nixon RA (2013). The role of autophagy in neurodegenerative disease. Nat. Med..

[36] Rodolfo, C., Campello, S., & Cecconi, F. Mitophagy in neurodegenerative diseases. *Neurochem. Int.* (2017), in press. 10.1016/j.neuint.2017.08.004 28797885

[37] Ransohoff RM (2016). How neuroinflammation contributes to neurodegeneration. Science (New York, NY).

[38] Chitnis, T., Weiner, H. L. CNS inflammation and neurodegeneration. *J .Clin. Invest*. **127**, 3577-3587 (2017). 10.1172/JCI90609 PMC5617655 28872464

[39] Kriz J, Nguyen M, Julien J (2002). Minocycline slows disease progression in a mouse model of amyotrophic lateral sclerosis. Neurobiol. Dis..

[40] Csordas G (2006). Structural and functional features and significance of the physical linkage between ER and mitochondria. J. Cell Biol..

[41] Filadi R, Theurey P, Pizzo P (2017). The endoplasmic reticulum-mitochondria coupling in heath and disease: molecules, functions and significance. Cell Calcium.

[42] van Vliet A, Verfaillie T, Agostinis P (2014). New functions of mitochondria associated membranes in cellular signalling. Biochim. Biophys. Acta.

[43] Rowland AA, Voeltz GK (2012). Endoplasmic reticulum-mitochondria contacts: function of the junction. Nat. Rev. Mol. Cell Biol..

[44] Helle SC (2013). Organization and function of membrane contact sites. Biochim. Biophys. Acta.

[45] Krols M, Bultynck G, Janssens S (2016). ER-mitochondria contact sites: a new regulator of cellular calcium flux comes into play. J. Cell Biol..

[46] Area-Gomez E (2012). Upregulated function of mitochondria-associated ER membranes in Alzheimer disease. EMBO J..

[47] Griffiths EJ, Rutter GA (2009). Mitochondrial calcium as a key regulator of mitochondrial ATP production in mammalian cells. Biochim. Biophys. Acta.

[48] Korobova F, Ramabhadran V, Higgs HN (2013). An actin-dependent step in mitochondrial fission mediated by the ER-associated formin INF2. Science (New York, NY).

[49] Lewis SC, Uchiyama LF, Nunnari J (2016). ER-mitochondria contacts couple mtDNA synthesis with mitochondrial division in human cells. Science (New York, NY).

[50] Friedman JR (2011). ER tubules mark sites of mitochondrial division. Science (New York, NY).

[51] Friedman JR, Webster BM, Mastronarde DN, Verhey KJ, Voeltz GK (2010). ER sliding dynamics and ER-mitochondrial contacts occur on acetylated microtubules. J. Cell Biol..

[52] Macaskill AF (2009). Miro1 is a calcium sensor for glutamate receptor-dependent localization of mitochondria at synapses. Neuron.

[53] Saotome M (2008). Bidirectional Ca2+-dependent control of mitochondrial dynamics by the Miro GTPase. Proc. Natl. Acad. Sci. USA.

[54] Kornmann B, Osman C, Walter P (2011). The conserved GTPase Gem1 regulates endoplasmic reticulum-mitochondria connections. Proc. Natl. Acad. Sci. USA.

[55] Wang X, Schwarz TL (2009). The mechanism of Ca2+-dependent regulation of kinesin-mediated mitochondrial motility. Cell.

[56] Hailey DW (2010). Mitochondria supply membranes for autophagosome biogenesis during starvation. Cell.

[57] Hamasaki M (2013). Autophagosomes form at ER-mitochondria contact sites. Nature.

[58] Garofalo, T. et al. Evidence for the involvement of lipid rafts localized at the ER-mitochondria associated membranes in autophagosome formation. *Autophagy*** 12**, 917-935 (2016). 10.1080/15548627.2016.1160971 PMC4922444 27123544

[59] Wu W (2016). FUNDC1 regulates mitochondrial dynamics at the ER-mitochondrial contact site under hypoxic conditions. EMBO J..

[60] Bockler S, Westermann B (2014). Mitochondrial ER contacts are crucial for mitophagy in yeast. Dev. Cell.

[61] Gomez-Suaga P (2017). The ER-mitochondria tethering complex VAPB−PTPIP51 regulates autophagy. Curr. Biol..

[62] Vicencio JM (2009). The inositol 1,4,5-trisphosphate receptor regulates autophagy through its interaction with Beclin 1. Cell Death Differ..

[63] Wong A, Grubb DR, Cooley N, Luo J, Woodcock EA (2013). Regulation of autophagy in cardiomyocytes by Ins(1,4,5)P(3) and IP(3)-receptors. J. Mol. Cell Cardiol..

[64] Khan MT, Joseph SK (2010). Role of inositol trisphosphate receptors in autophagy in DT40 cells. J. Biol. Chem..

[65] Criollo A (2007). Regulation of autophagy by the inositol trisphosphate receptor. Cell Death Differ..

[66] Cardenas C (2010). Essential regulation of cell bioenergetics by constitutive InsP3 receptor Ca2+ transfer to mitochondria. Cell.

[67] Sarkar S (2005). Lithium induces autophagy by inhibiting inositol monophosphatase. J. Cell Biol..

[68] Mallilankaraman K (2012). MCUR1 is an essential component of mitochondrial Ca2+ uptake that regulates cellular metabolism. Nat. Cell Biol..

[69] Cardenas C (2016). Selective vulnerability of cancer cells by inhibition of Ca transfer from endoplasmic reticulum to mitochondria. Cell Rep..

[70] Simmen T (2005). PACS-2 controls endoplasmic reticulum-mitochondria communication and Bid-mediated apoptosis. EMBO J..

[71] Simmen T, Lynes EM, Gesson K, Thomas G (2010). Oxidative protein folding in the endoplasmic reticulum: tight links to the mitochondria-associated membrane (MAM). Biochim. Biophys. Acta.

[72] Bravo R (2011). Increased ER-mitochondrial coupling promotes mitochondrial respiration and bioenergetics during early phases of ER stress. J. Cell Sci..

[73] Kanekura K, Nishimoto I, Aiso S, Matsuoka M (2006). Characterization of amyotrophic lateral sclerosis-linked P56S mutation of vesicle-associated membrane protein-associated protein B (VAPB/ALS8). J. Biol. Chem..

[74] Gkogkas C (2008). VAPB interacts with and modulates the activity of ATF6. Hum. Mol. Genet..

[75] Zhou R, Yazdi AS, Menu P, Tschopp J (2011). A role for mitochondria in NLRP3 inflammasome activation. Nature.

[76] Kornmann B (2009). An ER-mitochondria tethering complex revealed by a synthetic biology screen. Science (New York, NY).

[77] de Brito OM, Scorrano L (2008). Mitofusin 2 tethers endoplasmic reticulum to mitochondria. Nature.

[78] Naon D (2016). Critical reappraisal confirms that Mitofusin 2 is an endoplasmic reticulum-mitochondria tether. Proc. Natl. Acad. Sci. USA.

[79] De Vos KJ (2012). VAPB interacts with the mitochondrial protein PTPIP51 to regulate calcium homeostasis. Hum. Mol. Genet..

[80] Stoica R (2014). ER-mitochondria associations are regulated by the VAPB−PTPIP51 interaction and are disrupted by ALS/FTD-associated TDP-43. Nat. Commun..

[81] Gomez-Suaga P, Paillusson S, Miller CCJ (2017). ER-mitochondria signaling regulates autophagy. Autophagy.

[82] Huttlin EL (2015). The BioPlex network: a systematic exploration of the human interactome. Cell.

[83] Galmes R (2016). ORP5/ORP8 localize to endoplasmic reticulum-mitochondria contacts and are involved in mitochondrial function. EMBO Rep..

[84] Qiao X (2017). PTPIP51 regulates mouse cardiac ischemia/reperfusion through mediating the mitochondria-SR junction. Sci. Rep..

[85] Jain A, Beutel O, Ebell K, Korneev S, Holthuis JC (2017). Diverting CERT-mediated ceramide transport to mitochondria triggers Bax-dependent apoptosis. J. Cell Sci..

[86] Cho IT (2017). Ascorbate peroxidase proximity labeling coupled with biochemical fractionation identifies promoters of endoplasmic reticulum mitochondrial contacts. J. Biol. Chem..

[87] Hung V (2017). Proteomic mapping of cytosol-facing outer mitochondrial and ER membranes in living human cells by proximity biotinylation. eLife.

[88] Iwasawa R, Mahul-Mellier AL, Datler C, Pazarentzos E, Grimm S (2011). Fis1 and Bap31 bridge the mitochondria-ER interface to establish a platform for apoptosis induction. EMBO J..

[89] Stoica R (2016). ALS/FTD-associated FUS activates GSK-3beta to disrupt the VAPB-PTPIP51 interaction and ER-mitochondria associations. EMBO Rep..

[90] Llorens-Martin M, Jurado J, Hernandez F, Avila J (2014). GSK-3beta, a pivotal kinase in Alzheimer disease. Front. Mol. Neurosci..

[91] Lovestone S (1994). Alzheimer’s disease-like phosphorylation of the microtubule-associated protein tau by glycogen synthase kinase-3 in transfected mammalian cells. Curr. Biol..

[92] Israel MA (2012). Probing sporadic and familial Alzheimer’s disease using induced pluripotent stem cells. Nature.

[93] Bernard-Marissal N, Medard JJ, Azzedine H, Chrast R (2015). Dysfunction in endoplasmic reticulum-mitochondria crosstalk underlies SIGMAR1 loss of function mediated motor neuron degeneration. Brain.

[94] Watanabe S (2016). Mitochondria-associated membrane collapse is a common pathomechanism in SIGMAR1- and SOD1-linked ALS. EMBO Mol. Med..

[95] Luty AA (2010). Sigma nonopioid intracellular receptor 1 mutations cause frontotemporal lobar degeneration-motor neuron disease. Ann. Neurol..

[96] Al-Saif A, Al-Mohanna F, Bohlega S (2011). A mutation in sigma-1 receptor causes juvenile amyotrophic lateral sclerosis. Ann. Neurol..

[97] Ullah MI (2015). In silico analysis of SIGMAR1 variant (rs4879809) segregating in a consanguineous Pakistani family showing amyotrophic lateral sclerosis without frontotemporal lobar dementia. Neurogenetics.

[98] Gregianin E (2016). Loss-of-function mutations in the SIGMAR1 gene cause distal hereditary motor neuropathy by impairing ER-mitochondria tethering and Ca2+ signalling. Hum. Mol. Genet..

[99] Dreser A. et al. The ALS-linked E102Q mutation in Sigma receptor-1 leads to ER stress-mediated defects in protein homeostasis and dysregulation of RNA-binding proteins. *Cell Death Differ.* (2017), in press. 10.1038/cdd.2017.88 PMC5596426 28622300

[100] Hayashi T, Su TP (2007). Sigma-1 receptor chaperones at the ER-mitochondrion interface regulate Ca(2+) signaling and cell survival. Cell.

[101] Tagashira H, Bhuiyan MS, Shioda N, Fukunaga K (2014). Fluvoxamine rescues mitochondrial Ca2+transport and ATP production through sigma(1)-receptor in hypertrophic cardiomyocytes. Life Sci..

[102] Su TP, Su TC, Nakamura Y, Tsai SY (2016). The sigma-1 receptor as a pluripotent modulator in living systems. Trends Pharmacol. Sci..

[103] Belzil VV (2013). Genetic analysis of SIGMAR1 as a cause of familial ALS with dementia. Eur. J. Hum. Genet..

[104] Mancuso R (2012). Sigma-1R agonist improves motor function and motoneuron survival in ALS mice. Neurotherapeutics.

[105] Ono Y (2014). SA4503, a sigma-1 receptor agonist, suppresses motor neuron damage in in vitro and in vivo amyotrophic lateral sclerosis models. Neurosci. Lett..

[106] Hyrskyluoto A (2013). Sigma-1 receptor agonist PRE084 is protective against mutant huntingtin-induced cell degeneration: involvement of calpastatin and the NF-kappaB pathway. Cell Death Dis..

[107] Mavlyutov TA (2013). Lack of Sigma-1 receptor exacerbates ALS progression in mice. Neuroscience.

[108] Prause J (2013). Altered localization, abnormal modification and loss of function of Sigma receptor-1 in amyotrophic lateral sclerosis. Hum. Mol. Genet..

[109] Vollrath JT (2014). Loss of function of the ALS protein SigR1 leads to ER pathology associated with defective autophagy and lipid raft disturbances. Cell Death Dis..

[110] Nishimura AL (2004). A mutation in the vesicle-trafficking protein VAPB causes late-onset spinal muscular atrophy and amyotrophic lateral sclerosis. Am. J. Hum. Genet..

[111] Mitne-Neto M (2011). Downregulation of VAPB expression in motor neurons derived from induced pluripotent stem-cells of ALS8 patients. Hum Mol Genet.

[112] Anagnostou G (2010). Vesicle associated membrane protein B (VAPB) is decreased in ALS spinal cord. Neurobiol. Aging.

[113] Kim JY, Jang A, Reddy R, Yoon WH, Jankowsky JL (2016). Neuronal overexpression of human VAPB slows motor impairment and neuromuscular denervation in a mouse model of ALS. Hum. Mol. Genet..

[114] Paillusson S (2017). alpha-Synuclein binds to the ER-mitochondria tethering protein VAPB to disrupt Ca2+ homeostasis and mitochondrial ATP production. Acta Neuropathol..

